# Investigation and analysis of porcine epidemic diarrhea cases and evaluation of different immunization strategies in the large-scale swine farming system

**DOI:** 10.1186/s40813-023-00331-z

**Published:** 2023-08-03

**Authors:** Bingzhou Zhang, Jie Qing, Zhong Yan, Yuntong Shi, Zewei Wang, Jing Chen, Junxian Li, Shuangxi Li, Weisheng Wu, Xiaofang Hu, Yang Li, Xiaoyang Zhang, Lili Wu, Shouyue Zhu, Zheng Yan, Yongquan Wang, Xiaoli Guo, Ligen Yu, Xiaowen Li

**Affiliations:** 1 Shandong New Hope Liuhe Agriculture and Animal Husbandry Technology Co., Ltd. (NHLH Academy of Swine Research), Dezhou, 253034 China; 2 Xiajin New Hope Liuhe Agriculture and Animal Husbandry Co., Ltd, Dezhou, 253200 China; 3 Research Center of Information Technology, Beijing Academy of Agriculture and Forestry Sciences, Beijing, China; 4 China Agriculture Research System-Yangling Comprehensive test Station, Xianyang, 712100 China; 5 Shandong Engineering Laboratory of Pig and Poultry Healthy Breeding and Disease Diagnosis Technology, Qingdao, China

**Keywords:** PEDV, Prevalence, Immunization strategy, The duration of PED epidemic, Economic evaluation model

## Abstract

**Background:**

Porcine epidemic diarrhea (PED) is a contagious intestinal disease caused by porcine epidemic diarrhea virus (PEDV) characterized by vomiting, diarrhea, anorexia, and dehydration, which has caused huge economic losses around the world. However, it is very hard to find completely valid approaches to control the transmission of PEDV. At present, vaccine immunity remains the most effective method. To better control the spread of PED and evaluate the validity of different immunization strategies, 240 PED outbreak cases from 577 swine breeding farms were collected and analyzed. The objective of the present study was to analyze the epidemic regularity of PEDV and evaluate two kinds of different immunization strategies for controlling PED.

**Results:**

The results showed that the main reasons which led to the outbreak of PED were the movement of pig herds between different pig farms (41.7%) and delaying piglets from the normal production flow (15.8%). The prevalence of PEDV in the hot season (May to October) was obviously higher than that in the cold season (January to April, November to December). Results of different vaccine immunity cases showed that immunization with the highly virulent live vaccine (NH-TA2020 strain) and the commercial inactivated vaccine could significantly decrease the frequency of swine breeding farms (5.9%), the duration of PED epidemic (1.70 weeks), and the week batches of dead piglets (0.48 weeks weaned piglets), compared with immunization with commercial attenuated vaccines and inactivated vaccine of PED. Meanwhile, immunization with the highly virulent live vaccine and the commercial inactivated vaccine could bring us more cash flows of Y̶275,274 per year than immunization with commercial live attenuated vaccine and inactivated vaccine in one 3000 sow pig farm within one year.

**Conclusion:**

Therefore, immunization with highly virulent live vaccine and inactivated vaccine of PED is more effective and economical in the prevention and control of PED in the large-scale swine farming system.

## Introduction

Porcine epidemic diarrhea (PED) is caused by porcine epidemic diarrhea virus (PEDV) with symptoms including diarrhea, vomiting, anorexia, dehydration, and weight loss in piglets [1, 2]. PEDV was first identified in the 1980s in China, and since then, it has become one of the most common viral causes of diarrhea. In October 2010, a large-scale outbreak of PED caused by a PEDV variant occurred in China [1]. Pigs of all ages can be infected with different symptoms and the mortality in piglets is up to 100% [3], which has led to huge economic losses all around the world.

In the winter of 2010, PED outbreaks occurred in China which caused large economic losses [4]. Subsequently, in the spring of 2013, the first PED case was reported in the United States, which led to astonishing mortality from the winter of 2013 to 2014 and severely affect the pork industry [5, 6]. Chang et al. detected 1311 diarrhea samples from 8 provinces of China in 2021, the study concluded that the positive rate of four seasons from spring to winter is 71.79%, 36.88%, 48.43%, and 58.76%, respectively [7]. Based on the published PED cases, we found that PEDV outbreaks frequently often focused on the early spring and winter months in temperate regions, peaking between November and March, which was the same as the previous researches [8–11]. Therefore, environmental temperature control is very important to prevent PEDV infection in the cold seasons. In this study, we also analyze the seasonal characteristic of PED in the large-scale swine farming system.

Dang et al. found that the infection of PEDV usually spread among pigs of different ages in the following sequence: first infected was the fattening/replacement pigs; then the virus accumulated and infected pregnant sows, bringing the virus to the delivery room; the subclinically infected sows then transmitted PEDV to the suckling piglets, giving rise to an eventual epidemic among the piglets [1]. Therefore, it is very important to prevent fattening/replacement pigs from being infected and cut off the spread from subclinically infected sows to suckling piglets in controlling PED. Strict biosecurity measures are also very effective in protecting uninfected pigs [12].

At present, the most common method used in controlling PED is vaccine immunization, including commercial live attenuated vaccines and commercial inactivated vaccines. Vaccination of sows before farrowing induces lactogenic immunity, which is transferred to neonatal piglets via colostrum [13]. Commercial vaccines provide only partial protection, they cannot completely block suckling piglets from infecting by PEDV [14]. Inactivated vaccines are safe but have a short duration of immunity and require the appropriate adjuvants for strong immune responses [15–17]. Live-attenuated vaccines, produced by serially passaging field strains, are more effective against homologous strains but have a long lead development time and cannot supply enough protection to highly virulent heterologous strains [18, 19]. Therefore, it is urgent to find a kind of novel vaccine or measures that confer better protection.

In this study, high virulent isolation strain (NH-TA2020 strain) was used to prevent the outbreak of PED, and different immunization strategies in the large-scale swine farming system were evaluated. To directly understand the difference between highly virulent live vaccines and commercial live attenuated vaccines, a new economic evaluation model of PED outbreak farms was constructed.

## Materials and methods

### Classification of swine herds by porcine epidemic diarrhea status

The location of swine farms from the large-scale swine farming system was distributed throughout almost the whole Chinese mainland. The number of sows in those swine farms was from 1000 to 3000. To better understand the PED status of the large-scale swine farming system, all swine farms were defined as four types based on shedding status, antibody level, and clinical symptoms of piglets and sows [20]. The RT-qPCR (PEDV-N gene) method and commercial kits (PEDV-S1 gene, IDEXX) were used to detect shedding status and evaluate PEDV-IgA antibody level of colostrum, respectively [21]. All samples were detected using the same testing procedures. The PED status of all swine farms was collected every month since January 2021. Detailed definitions of those four types of swine farms are as follows (Table 1).

**Table 1:** Classification of swine herds by porcine epidemic diarrhea status

Status	Antigen and antibody status	Clinical symptoms
PED Outbreak	Antigen: Positive Antibody: Positive	Number of infected piglets in the unit > 15% Severe clinical symptoms of PED, piglets death Vaccinated
PED Active	Antigen: Positive Antibody: Positive	Number of infected piglets in the unit < 15% Mild clinical symptoms of PED, no piglets death Vaccinated
PED Stable	Antigen: Negative Antibody: Positive	No clinical symptoms of PED, no piglets death Antigen: negative Vaccinated
PED Negative	Antigen: Negative Antibody: Negative	Swine farm: no PED history No vaccine

### Immunization strategies of sows in the large-scale swine farming system

All swine in the large-scale swine farming system were vaccinated with two kinds of strategies, and the same immunization strategy was used on the same farm. The one immune commercial live attenuated vaccine (WH-1R and AJ1102-R strains), and commercial inactivated vaccine (WH-1 and Strain AJ1102 strains) three weeks after the first dose of immunization (Group A). The other one immune highly virulent live vaccine (NH-TA2020 strain, isolated by Swine Research Institute of New Hope Group) by oral immunization, and the same commercial inactivated vaccine with the first strategy three weeks after the first dose of immunization (Group B) [21]. The other additional controlling measures are the same in these two kinds of swine farming systems. The detailed grouping results were presented in Table 2.

**Table 2:** Grouping results of different immunization strategies

Group	Immunization strategies
A	commercial live attenuated vaccine and commercial inactivated vaccine
B	highly virulent live vaccine and commercial inactivated vaccine

### Establishment of porcine epidemic diarrhea monitoring system

To monitor the PED status of all swine farms in the large-scale swine farming system, the following information was collected every week: the name of swine farms, number of sows, the average number of weaned piglets in one week, immune background, start time of “PED Outbreak”, end time of “PED Outbreak”, the number of dead piglets in one week, the causes of PED. To better evaluate the effect of different immunization strategies, two new definitions were introduced as follows:

Duration of PED epidemic (week) = (start time of “PED Outbreak” - end time of “PED Outbreak”) / 7.

The end time of “PED Outbreak” means that the number of infected piglets in the newborn delivery unit within 7 days < 15% of all piglets in the same unit and no piglets die from diarrhea caused by PEDV.

Week batch of dead piglets = the number of dead piglets / average number of weaned piglets in one week.

### Statistical analysis

All data were obtained from the large-scale swine farming system, and they were all credible. Results were presented as the means ± standard deviation (SD). The statistical analysis was performed using two-tailed *t*-tests in Graph Pad Prism 7.0 (GraphPad Software Inc., USA). The significant difference was defined as ∗ *p* < 0.05, and the various degrees of significant difference were designated as ∗∗ *p* < 0.01.

## Results

### The causes analysis of PED cases in the large-scale swine farming system

**Fig. 1:**
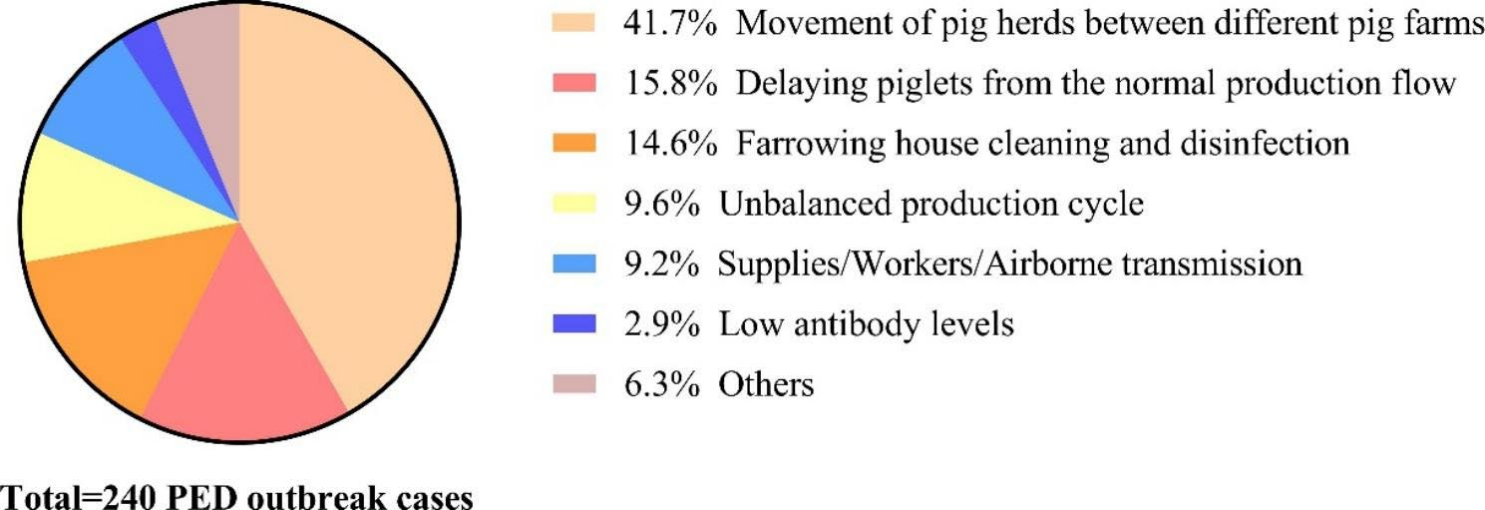
The causes analysis of PED cases in the swine farming system. The data from 240 PED cases were analyzed. The proportion of these causes was calculated respectively

The causes of all PED outbreaks in swine farms were investigated from January 2021 to December 2021 in all farming systems. The main causes of 240 PED cases from 176 PED outbreak swine farms were analyzed. The results showed that the cause ranking first was the movement of pig herds between different pig farms, which contained 100 PED cases, and the proportion reached 41.7%. The following causes were delaying piglets from the normal production flow (15.8%), farrowing house cleaning and disinfection (14.6%), unbalanced production cycle (9.6%), supplies/workers/airborne transmission (9.2%), low antibody levels (2.9%), and others (6.3%), respectively (Fig. 1). The detailed data were as follows:

### The seasonal prevalence characteristics of PEDV in the swine farming system

**Fig. 2:**
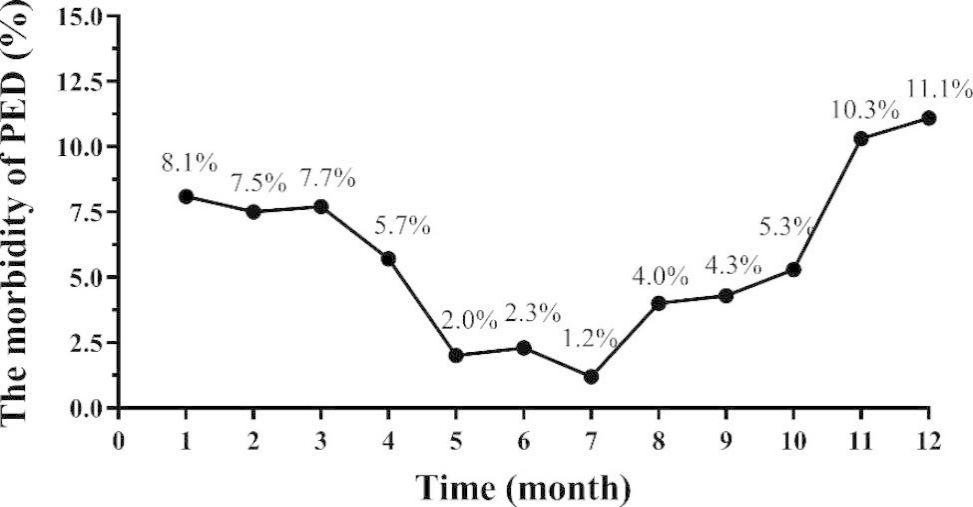
The seasonal prevalence of PEDV in 2021. The accurate prevalence from January to December was 8.1%, 7.5%, 7.7%, 5.7%, 2.0%, 2.3%, 1.2%, 4.0%, 4.3%, 5.3%, 10.3%, and 11.1%, respectively

To investigate the seasonal prevalence characteristics of PEDV, the number of PED outbreak farms were calculated from January to December 2021. Results showed that the prevalence of PEDV across the whole year ranged from 1.2 to 11.1% with the highest prevalence recorded in December and the lowest prevalence recorded in July. Therefore, the prevalence of PEDV in the hot season (May to October) was obviously higher than that in the cold season (January to April, November to December) (Fig. 2). The epidemic trend of PEDV in different months had no significant difference between the swine using different immunization strategies (highly virulent live vaccine and commercial inactivated vaccine/commercial live attenuated vaccine and inactivated vaccine).

### PED outbreak frequency analysis of 176 PED outbreak farms

Statistical results showed that 176 swine farms were affected by PED in 577 investigated swine farms from January 2021 to December 2021. Among them, there were 240 PED cases happened, and the morbidity for the whole of 2021 reached 30.7%. To better understand the frequency of investigated swine farms, detailed information about every swine farm was recorded. Results revealed that there were 47 swine farms affected by PED more than one time, and the highest frequency reached 4 times within one year.

**Fig. 3:**
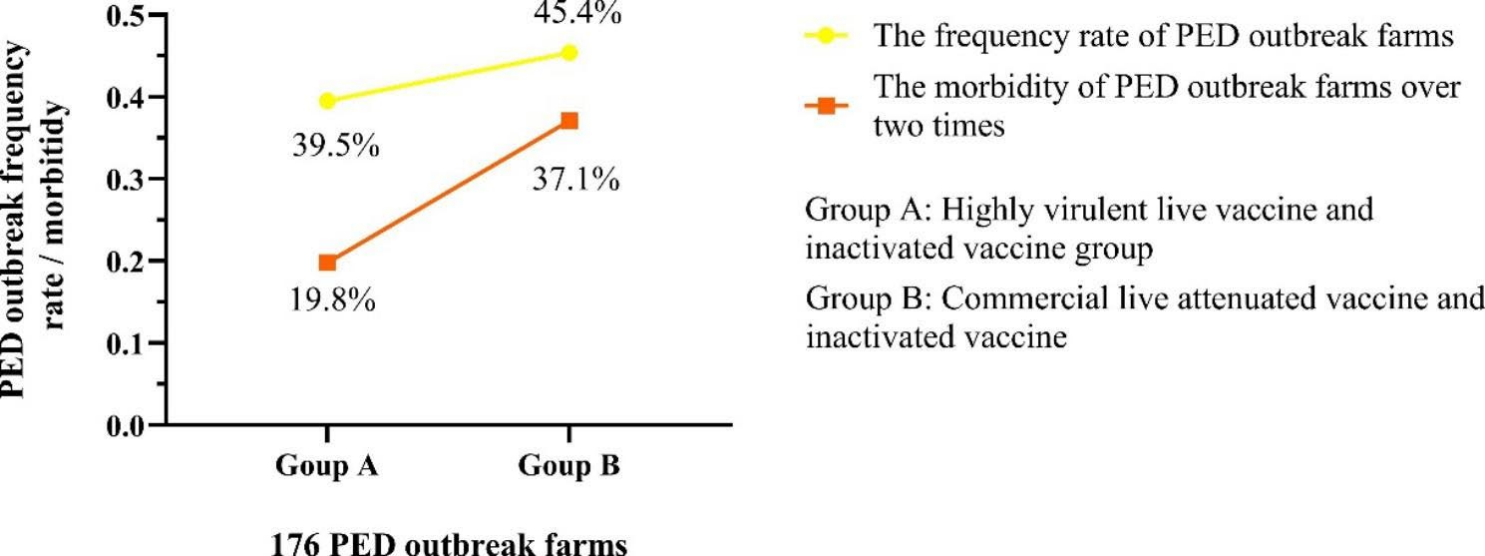
The frequency of 176 PED outbreak farms and the morbidity of PED outbreak farms over two times. The proportion of frequency and morbidity of 176 PED outbreak farms were calculated respectively

By comparing the immune background of these PED outbreak farms, we found that the swine vaccinated with the highly virulent live vaccine (NH-TA2020 strain) and the commercial inactivated vaccine had significantly lower PED outbreak frequency (146/370, 39.5%) compared with PED outbreak frequency of the swine vaccinated with commercial live attenuated vaccine and inactivated vaccine (94/207, 45.4%) (Fig. 3). Similar results in these PED outbreak farms over 2 times were also found. The proportion of these PED outbreak farms over 2 times vaccinated with the highly virulent live vaccine (NH-TA2020 strain) and commercial inactivated vaccine (21/106, 19.8%) was obviously lower than the proportion of PED outbreak farms over 2 times vaccinated with commercial live attenuated vaccines and inactivated vaccine (26/70, 37.1%) (Fig. 3).

### The duration of PED epidemic with different immunization strategies

To evaluate the effectiveness of PED prevention and control measures, the duration of PED epidemic with different immunization strategies was analyzed. The results indicated that the average duration of all PED outbreak farms was 4.15 weeks. However, the duration of the PED epidemic vaccinated with commercial live attenuated vaccine and inactivated vaccine was 5.18 weeks, which was obviously higher than that of PED outbreak farms vaccinated with highly virulent live vaccine and commercial inactivated vaccine (3.48 weeks) (Fig. 4A).

**Fig. 4A:**
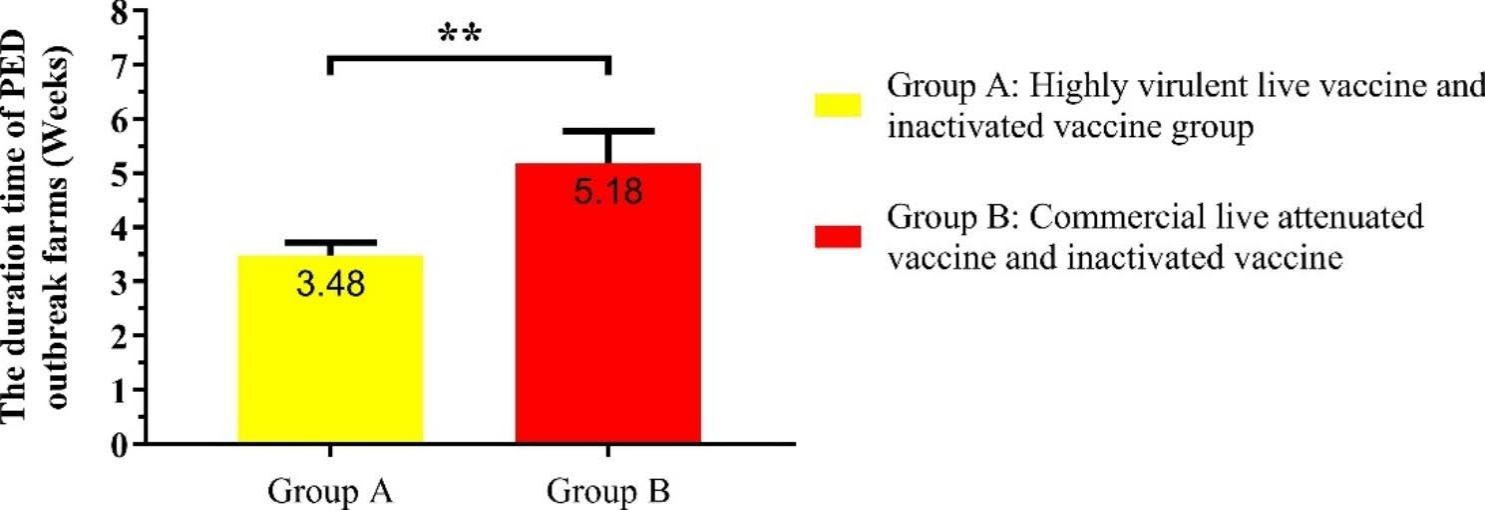
The duration of PED epidemic with different immunization strategies. All data from the PED outbreak farms in the swine farming system. Bars represent the mean ± standard error. Statistical analyses were performed using two-way ANOVA. ** represents significant difference (*p* < 0.01)

On the other hand, the swine farms where the PED epidemic over 4 weeks were also analyzed. Analysis results indicated that the proportion of PED outbreak farms vaccinated with highly virulent live vaccine and the commercial inactivated vaccine was 10.8% (40/370), which also had a significant difference with PED outbreak farms vaccinated with commercial live attenuated vaccine and inactivated vaccine (32/207, 15.5%) (Fig. 4B).

**Fig. 4B:**
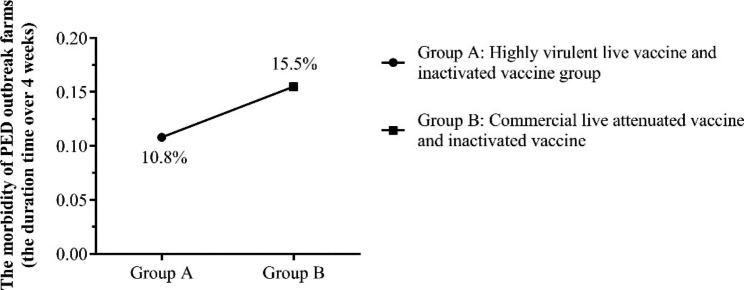
The morbidity of PED outbreak farms that the PED epidemic over 4 weeks with different immunization strategies

### The week batches of dead piglets in PED outbreak farms with different immunization strategies

The week batches of dead piglets were also seriously affected by PED. The results showed that the average week batches of dead piglets of all PED outbreak farms were 1.43 weeks. However, the average week batches of dead piglets of PED outbreak farms vaccinated with commercial live attenuated vaccine and inactivated vaccine was 1.72 weeks, which was obviously higher than PED outbreak farms vaccinated with highly virulent live vaccine and commercial inactivated vaccine (1.24 weeks) (Fig. 5A).

**Fig. 5A:**
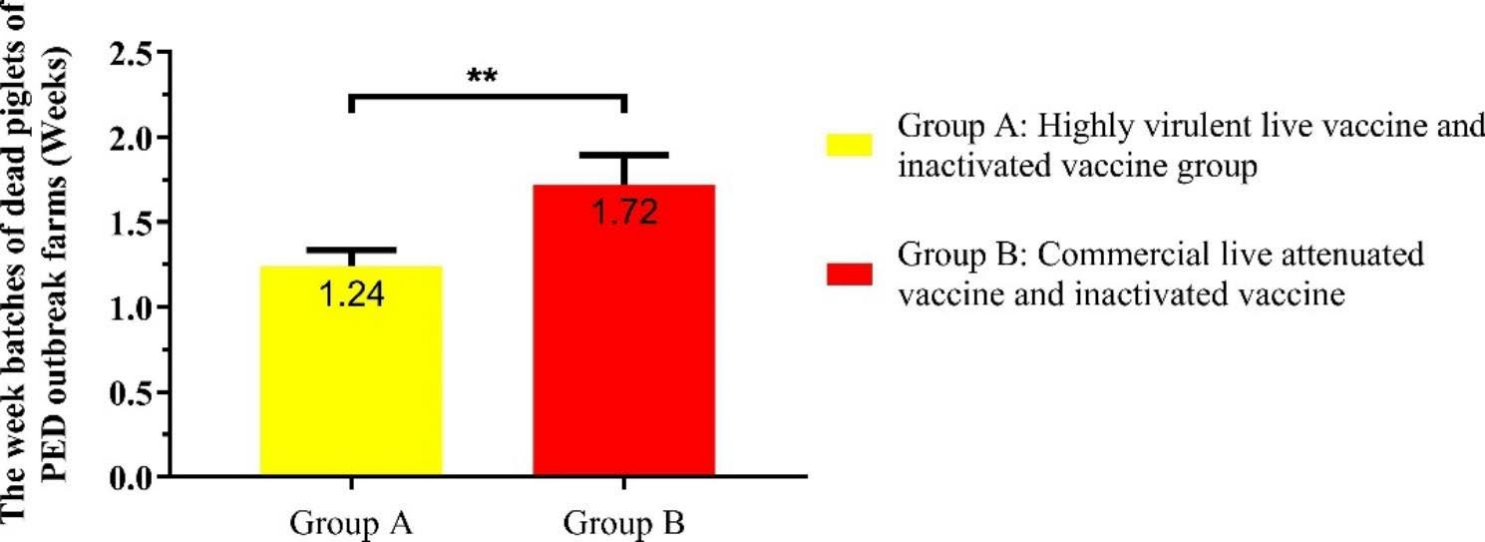
The week batches of dead piglets of PED outbreak farms with different immunization strategies. All data from the PED outbreak farms in the swine farming system. Bars represent the mean ± standard error. Statistical analyses were performed using two-way ANOVA. ** represents significant difference (*p* < 0.01)

Meanwhile, the swine farms with the week batches of dead piglets over 1.5 weeks were also analyzed. Analysis results showed that the proportion of PED outbreak farms vaccinated with highly virulent live vaccine and the commercial inactivated vaccine was 11.6% (43/370), which was obviously lower than PED outbreak farms vaccinated with commercial live attenuated vaccine and inactivated vaccine (33/207, 15.9%) (Fig. 5B).

**Fig. 5B:**
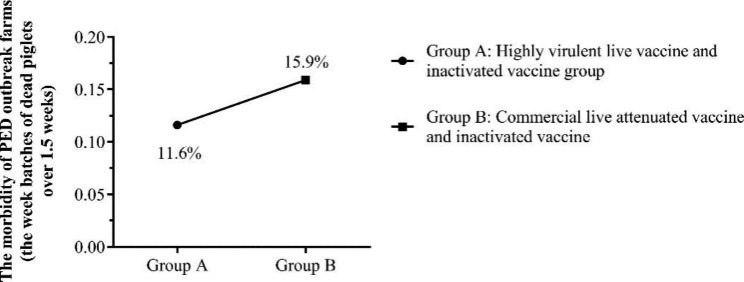
The morbidity of PED outbreak farms with the week batches of dead piglets over 1.43 weeks

### The economic evaluation model of PED outbreak farms

For a farm kept for profit, the decision on immunization strategies was not only based on the duration and morbidity of the PED outbreak but also, or even prominently on the economic outcome. Therefore, we conducted a profit analysis.

Here, we focused on three indicators mentioned above, i.e., the duration, morbidity, and frequency of the PED outbreak, and the outcome of weaning piglets in our economic analysis. Assuming piglets of various health conditions with the same price per kilogram, average prices (considering CPI) of piglets during 2011–2020 in China were used and the difference between cash inflows that the sales of piglets could bring us and that was reduced by the PED vaccine costs were compared. In this study, We used the basic data to calculate the cash inflows of a farm with the size of 3000-sow, 7-day production rhythm, and assume only the vaccines used and amount of piglets were different, all else equal. The results showed that immunization with the highly virulent live vaccine and the commercial inactivated vaccine can bring us more cash flows of Y̶275,274 per year than immunization with commercial live attenuated vaccine and inactivated vaccine (Table 3).

**Table 3:** The economic evaluation model of PED outbreak farms

Details	Normal production	Group B	Group A
The average amount of weaned piglets per week per sow	11.6		
Number of farrowing sows per week	120		
The expected times of PED outbreak per year	/	0.39	0.45
The expected loss of piglets (weeks)	/	1.24	1.72
Piglets survival per year	0	-681	-1,087
Price of highly virulent live vaccine(Y̶/pig)	/	8	8
Price of commercial live attenuated vaccine(Y̶/pig)	/	11	11
Price of commercial inactivated vaccine(Y̶/pig)	/	11	11
The 10-year average price of piglets/kg in China(Y̶)	38.49	38.49	38.49
Duration of PED epidemic(weeks)	/	3.48	5.18
Average piglet weight during PED epidemic	/	3.97	3.85
Normal piglet weight	6	6	6
Number of normal piglets	0	-4,844	-7,211
Number of abnormal piglets	0	4,163	6,123
Cash inflows	0	-538,732.70	-814,007.09
Difference between group A and group B		275,274.39	

## Discussion

To better understand the PED status of investigated swine farms, they were divided into four types, “PED Outbreak”, “PED Active”, “PED Stable”, and “PED Negative”. The information of all investigated swine farms was collected. Meanwhile, the data of all PED outbreak farms were analyzed, which included the reasons that lead to the outbreak of PED, seasonal prevalence characteristics of PED, PED outbreak frequency, the duration of PED epidemic, the week batches of dead piglets in PED outbreak farms, and economic losses of two kinds of immunization strategies, respectively.

In our research, the movement of pig herds between different pig farms was the main reason that led to the outbreak of PED. According to the reports, subclinically infected sows were the main sources of PEDV, which could transmit PEDV to the suckling piglets from the fecal-oral route and colostrum-oral route [1, 8, 22]. Therefore, maybe some subclinically infected sows existed in our transferred pig herds, which led to the circulating transmission of PEDV in new swine farms. On the other hand, Indirect contact transmission of PEDV was also frequent within and between farms, particularly, with low biosecurity, via other contaminated fomites [12]. The fecal-nasal route was another route of pig-to-pig or farm-to-farm (up to 10 miles away) airborne transmission of PEDV via aerosolized PEDV particles that were infectious in nursing pigs [23–26]. Those all were the reasons for frequent PED outbreaks in swine farms.

Detecting results of diarrhea samples from 2018 to 2021 found that the positive rate of PEDV reached the highest in spring and winter, while it was relatively low in the other two seasons, with the lowest level in summer [7]. Chen et al. also found that the prevalence of PEDV in spring (50%) and winter (55%) was also much higher than that in summer (16%) and autumn (31%) [10]. In the current study, the prevalence of PEDV infection from January to December was 8.1%, 7.5%, 7.7%, 5.7%, 2.0%, 2.3%, 1.2%, 4.0%, 4.3%, 5.3%, 10.3%, and 11.1%, respectively, which was similar with previous researches [7, 10]. The lower critical temperature for PEDV survival may explain the seasonal variation in PEDV infection rates. These findings also suggested that efforts towards the prevention and control of PED should be most focused on in winter and spring.

A previous study showed that sows in outbreak herds were orally exposed to PEDV virulent strain contributed to the prevention and control of PED, and the sows could deliver healthy live-born pigs 3–4 weeks after oral exposure [27]. Meanwhile, traditional vaccines of PED were often limited in protecting suckling piglets from PEDV, including commercial live attenuated vaccines and inactivated vaccines [14]. The main prevalent PEDV strain in our swine farming system was GIIc types [21]. However, the traditional vaccine (WH-1R and AJ1102-R strains) that we used in the swine herd was designed based on the GIIb PEDV strain, and the cross-protection between different serotype strains was not very good [28]. These were all very important reasons that traditional vaccine could not show good performance in protecting piglets. Research also found that the colostrum containing high levels of IgA antibody induced by the NH-TA2020 strain could protect piglets against challenge by PEDV [21]. Therefore, the new variant PEDV strain (NH-TA2020) was used. However, the negative effects caused by PED in swine farms were often very complicated and hard to evaluate. In this study, three main targets were chosen to investigate the difference between two kinds of immunization strategies, including the frequency of PED outbreak farms, the duration of PED epidemic, and the week batches of dead piglets. Experimental results proved that the swine vaccinated with the highly virulent live vaccine (NH-TA2020 strain) and the commercial inactivated vaccine had significantly lower PED outbreak frequency, shorter average duration of PED epidemic, and fewer week batches of dead piglets in PED outbreak farms, compared with PED outbreak frequency of the swine vaccinated with commercial live attenuated vaccine and inactivated vaccine. Therefore, the new immunization strategy was more effective in the prevention and control of PED.

Research showed that the estimated annual costs of a PED outbreak with the closure of the breeding herd as the only intervention was approximately $300,000 for a 700-sow farrow-to-finishing herd. The most profitable strategy was the feedback of infected material and intensive biosecurity protocols, which could reduce 90% losses [29]. Jung-Da also found that the outbreak of PEDV caused an increase in the rate of NPDs in breeding herds, which also led to indirect economic loss [30]. Those researches often focused on comparing the loss of different measures in controlling PED. However, seldom researches focus on the prevention of PED. In this study, two kinds of immunization strategies were compared using a simple and efficient economic evaluation model. The study showed that immunization with the highly virulent live vaccine and the commercial inactivated vaccine can reduce losses by about Y̶275,274 per year than immunization with commercial live attenuated vaccine and inactivated vaccine. Meanwhile, some factors were not taken into consideration, such as nonproductive days in the breeding herd, labor charges, costs for breeding, feed, water, veterinary, disposal, and transport in the breeding unit [31, 32]. Moreover, we recommend the analysis to discuss different dimensions, for example, morbidity in different seasons or areas, which distinguish the profits. Details of sows and weaned piglets are also valuable, including post-outbreak production performance of sows and weaning weight of piglets, etc… In addition, prominent indicators, such as TTBP and the change of weaning weight and number of piglets should be considered.

## Conclusion

PED has caused tremendous piglet losses all around the world. Meanwhile, different prevention and control measures have been taken in reducing economic losses. However, seldom researches focus on the prevention of PED. In this study, two kinds of immunization strategies were compared. The results showed that immunization with the highly virulent live vaccine and the commercial inactivated vaccine can significantly reduce the frequency of swine breeding farms, shorten the duration of PED epidemic, decrease the week batches of dead piglets, and reduce economic loss, compared with immunization with commercial attenuated vaccines and inactivated vaccine of PED. Therefore, highly virulent live vaccines played an important role in the prevention of PED.

## Data Availability

All data generated or analyzed during this study are included in this published article.
